# The association between hematological markers of inflammation and chronic cannabis use: a systematic review and meta-analysis of observational studies

**DOI:** 10.3389/fpsyt.2024.1438002

**Published:** 2024-10-22

**Authors:** Reza Moshfeghinia, Amirhossein Najibi, Mehrnaz Moradi, Kasra Assadian, Jamshid Ahmadi

**Affiliations:** ^1^ Student Research Committee, Shiraz University of Medical Sciences, Shiraz, Iran; ^2^ Research Center for Psychiatry and Behavioral Sciences, Shiraz University of Medical Sciences, Shiraz, Iran; ^3^ Substance Abuse Research Center, Shiraz University of Medical Sciences, Shiraz, Iran; ^4^ Fasa Neuroscience Circle (FNC), Student Research Committee, Fasa University of Medical Sciences, Fasa, Iran; ^5^ Institute for Multicultural Counseling & Education Services (IMCES), Los Angeles, CA, United States

**Keywords:** neutrophil to lymphocyte ratio, NLR, cannabis, neuroinflammation, addiction

## Abstract

**Introduction:**

Cannabinoids, both natural and synthetic, are a subject of scientific interest. Cannabis is widely used, and its impact on health and the immune system is being studied. The endocannabinoid system influences inflammation, including the Neutrophil-to-Lymphocyte Ratio (NLR), a potential diagnostic tool. Our study investigates the connection between cannabis use and NLR.

**Methods:**

Our systematic review was registered in Prospero (#CRD42023463539). We searched six databases (PubMed, Scopus, Embase, PsycINFO, Web of Science, and CINAHL Complete) for records in English from inception to May 2024. We included observational studies that measured the Neutrophil-to-Lymphocyte Ratio (NLR) in cannabis users and control participants. We used the Newcastle–Ottawa Quality Assessment Scale to assess the quality of the included studies. We selected a random-effects model, and the statistical analysis was performed using Stata software version 17.

**Results:**

Out of a total of 4,054 records, only five articles were selected for inclusion in the meta-analysis. All of these chosen studies utilized a retrospective design. Furthermore, it's worth noting that all of the studies included were of high quality. In five studies involving 3,359 cannabis users and 10,437 non-users, no significant difference in NLR was found (WMD: 0.12 [-0.16, 0.41], I2: 39.89%). Subgroup analysis on healthy and schizophrenia participants didn't show significant NLR differences (p=0.76). Secondary analysis revealed cannabis users had higher Platelet-to-Lymphocyte Ratio (PLR) (67.80 [44.54, 91.06]), neutrophil count (0.68 [0.25, 1.12]), white blood cell count (0.92 [0.43, 1.41]), monocyte count (0.11 [0.05, 0.16]), and Systemic Immune Inflammation Index (SII) (83.48 [5.92, 157.04]) compared to non-users

**Conclusion:**

Our systematic review and meta-analysis reveal that cannabis use may affect NLR and hematologic parameters, suggesting a potential immune impact. Complex associations exist, requiring further research. Schizophrenia and pro-inflammatory factors are discussed, highlighting the need for ongoing investigation into cannabis-related immune changes and mental health.

**Systematic review registration:**

https://www.crd.york.ac.uk/prospero/, identifier CRD42023463539.

## 1 Introduction

Cannabis sativa, also known as marijuana, is the most widely used psychoactive substance globally, with an estimated 219 million users in 2021, according to the World Drug Report (1). Additionally, 27% of the North American population reported using cannabis for medical purposes (2). Cannabinoids are an emerging area of study showing potential for managing chronic pain, chemotherapy-induced nausea, and other medical conditions. Given its widespread use, the systemic effects warrant special attention (3–5). Cannabis may contribute to pulmonary symptoms, myocardial infarction, reduced spermatogenesis, and nonseminoma testicular cancer, besides its bidirectional relationship with psychiatric disorders such as psychosis, bipolar disorder, and schizophrenia (6–8).

Cannabinoids, which are botanical components of cannabis, can be categorized as synthetic or natural (9). They interact with the endocannabinoid system, modulating inflammation via G-coupled cannabinoid receptor 1 and, to a greater extent, receptor 2 (10). A systematic review of *in vitro* studies concluded that cannabinoids, especially cannabidiol and cannabigerol, can reduce inflammation by affecting inflammatory cytokines (11). Additionally, a study has shown that cannabinoids have anti-inflammatory effects in HIV patients' blood and CSF, which can be applied to HIV-related neurologic dysfunction (12).

Inflammatory biomarkers, derived from complete cell count (CBC) components, include the neutrophil-to-lymphocyte ratio (NLR), platelet-to-lymphocyte ratio (PLR), systemic immune-inflammation-index (SII), basophile-to-lymphocyte ratio (BLR), and monocyte to lymphocyte ratio (MLR) (13, 14). These CBC-derived inflammatory markers are convenient and available parameters for healthcare providers and are being investigated as biomarkers of substance use disorders. One study found that lymphocyte and monocyte levels were higher among non-opioid drug users compared to the healthy group, while platelet levels, NLR, PLR, and SII were higher in opioid drug users compared to non-opioid drug users (15). Another investigation revealed that NLR and MLR were higher in individuals with alcohol use disorder, but SII remained unaltered (14).

NLR is a reliable marker that reflects the dynamic relationship between the innate and adaptive immune systems and can be influenced by various infectious and non-infectious factors (16). NLR has been widely used and proposed as a diagnostic and prognostic tool. For instance, A higher NLR is associated with a higher mortality rate in patients with acute heart failure (17). Another study by Heather E. Soder et al. demonstrated an elevation in NLR among cocaine abusers (18). Evidence also suggests that cannabis cessation is associated with increases in leukocyte, monocyte, and lymphocyte levels, which correlate with psychosis symptoms post-cessation (19). NLR has been shown to increase in schizophrenia patients, both in the first episode of psychosis and in chronic disease, as demonstrated by Vasilios Karageorgiou et al. (20).

In this present systematic review and meta-analysis, we aim to investigate the correlation between cannabis use and inflammatory parameters within the CBC, specifically NLR. Our objective is to provide insight into the relationship between cannabis consumption and NLR levels. We seek to determine whether hematological markers of inflammation are altered in cannabis use disorder.

## 2 Methods

This systematic review and meta-analysis followed the Preferred Reporting Items for Systematic Reviews and Meta-Analyses (PRISMA) guidelines 2020 (21). The registration number in PROSPERO is CRD42023463539.

### 2.1 Search strategy

Five electronic databases (PubMed, Scopus, PsycINFO, Web of Science, and CINAHL Complete) were systematically queried for English-language records from their inception to May 2024. The searches were conducted utilizing keyword combinations such as "cannabis" OR "marijuana" AND "NLR" OR "Neutrophil to Lymphocyte Ratio." No synonyms or related terms were excluded from the search strategy. Detailed search strategies for each database can be found in **Supplementary Material 1**. Additionally, the references of the included studies were screened to identify potentially eligible articles.

### 2.2 Eligibility criteria

We incorporated observational research examining the impact of cannabis consumption on pro-inflammatory markers. Based on PECO, the following inclusion criteria were assessed:

Population and Exposure: patients with chronic use of synthetic or natural cannabis

Comparison: healthy control

Outcomes: The pro-inflammatory markers we assessed encompassed NLR, PLT, SII, and other hematological parameters.

Furthermore, we excluded studies following these criteria (1): Inadequate data for quantifying variances in pro-inflammatory indicators between subjects and control groups (2). Replication of studies or sharing participant data (3). Studies categorized as reviews, editorials, conference papers, case series/reports, secondary analyses, or animal experiments (4). Studies employing qualitative research methodologies.

### 2.3 Study selection

Two authors (AN and MM) autonomously reviewed the titles and abstracts of potentially eligible studies using EndNote. For studies deemed potentially eligible, separate authors independently assessed the full texts. Any conflicts concerning study design or methods, as well as the ultimate decision on whether to include studies, were resolved through a consensus meeting (RM).

### 2.4 Data extraction

Two authors, AN and MM, independently extracted information from the included articles. Any discrepancies were resolved through additional discussions. The following general characteristics were gathered: author names and publication years, study location, study design, sample size, ethnicity, male-to-female ratio, and the primary findings of the included studies (**Tables 1**, **2**).

**Table 1:** General characteristics of included studies.

Author	Country	Design	Type of cannabinoid exposure	Participants	Ethnicity	Male (%)	NLR, Mean (SD)	NOS score
**Guzel, et al., 2017 (** 22)	Turkey	Cross sectional	Synthetic, chronic use^*^	80 otherwise healthy subjects (40 user and 40 non-user)	Not mentioned	95% in both users and non-users	2.25 (0.99) in users VS 1.81 (0.61) in non-users	Good
**Goetz, et al., 2019 (** 23)	Georgia	Cross sectional, within subject design	No reference to synthetic or natural, any history of cannabis use	18 subjects with marijuana use and 43 non-user controls	White (27.8% VS 30.2) and African descent (72.2% VS 69.8)	61.1 of the users VS 62.8 of the non-users	2.09 (3.6) in users VS 2.25 (2.71) in non-users	Good
**Örüm, et al., 2020 (** 24)	Turkey	Cross sectional	no reference to synthetic or natural, Chronic use	56 patients with marijuana use disorder and 56 healthy adults as control cohort	Not mentioned	100 in both	2.3 (1.61) in users VS 2.43 (2.74) in non-users	Good
**Fridman, et al., 2023 (** 25)	Israel	Cross sectional	Synthetic, chronic use	144 SZ patients (110 users vs 34 non-users)	Israeli (no more details specified)	90 of the users and 70.9 of non-users	2 (1.18) in users VS 1.89 (0.82) in non-users	Good
**Al Hassan et al., 2023** (26)	United States of America	Cross sectional	No reference to synthetic or natural, Current use^**^	3211 current users VS 10213 never users of marijuana	Non-Hispanic white (62.1% current user VS 51.5% never user), Non-Hispanic Black (16.9% current user VS 11.5% never user), Hispanic and Mexican (13.5% current user VS 24.8% never user), Non-Hispanic Asian (2.1% current user VS 9.7% never user), and other race(5.4% current user VS 2.5% never user)	62.8 of current users VS 43.7 of never users	2.1(4.33) in current users VS 2.1(2.58) in never users	Good

*Defined per study protocol.

**Defined as self-reported marijuana use in the past 30 days.

**Table 2:** Main findings and features of included studies.

Author	Defining of groups	Method used for NLR measurement	Main findings	Limitations	Pathophysiology
**Guzel, et al., 2017 (** 22)	40 subjects diagnosed with cannabis use disorder according to DSM-V and without any other active or chronic disease as case and 40 healthy volunteers as control	Venous blood was drawn at the first day of hospitalization and stored in EDTA tubes. NLR was calculated by rating neutrophil count to lymphocyte count.	Statistically significant differences were observed between the two groups in terms of WBC, MCH, RDW, MCV, MPV, and NEU, LYM%, MONO%, IUBC, TIBC, and NLR parameters. Except for MPV and LYM which were lower in cannabis users, other parameters were significantly higher.	Small sample size Although smoking was eliminated as a confounding factor in this study, other confounding variables such as lifestyle and dietary characteristics were not accounted for.	Higher MCV in cannabis users could be a sign of Vitamin B12 and folic acid deficiency.
**Goetz, et al., 2019 (** 23)	Eighteen subjects with marijuana use, 24 subjects with cocaine use, and 43 subjects with a negative UDS met study inclusion/exclusion criteria. All patients had schizophrenia.	Results from CBC obtained within 48h of admission were extracted from digital medical records. NLR was not directly calculated in this study.	Patients with schizophrenia had not significantly higher total WBC, lymphocytes, and monocytes during hospitalizations with (vs. without) cannabis use.	Ratings of psychopathology were not available. This makes it difficult to adjust for psychosis severity as a potential confounding factor. No quantification of substance use No standard time of blood sample drawing Retrospective design The study was conducted on acutely ill SCZ patients.	No pathophysiologic explanations provided.
**Örüm, et al., 2020 (** 24)	56 male opium use disorder patients, 56 male marijuana use disorder patients and 56 healthy adult controls were studied. None of the participants had any history of psychiatric disorders.	CBC of patients and healthy controls were obtained from venous samples drawn from antecubital vein between 8 and 10 a.m. after at least 8 hours of fasting.	NLR and BLR differences were insignificant. Monocyte percentage was significantly lower in marijuana use disorder compared to control group. The optimal cutoff value for MONO was 0.55, and its sensitivity and specificity for diagnosis of MUD were 64.3% and 48.1%, respectively.	No laboratory measurement for confirmation of exposure Small sample size and not accounting for sex differences in the sample Not adjusting for confounding variables such as smoking, lifestyle and dietary characteristics, and age. Lack of understanding of the possible underlying mechanisms	Previous studies have shown the capacity of cannabinoids to decrease the number of T and B lymphocytes and increase eosinophils by modulation of cannabinoid receptor 2(CB2). In the Orum et al. study, Monocyte count was affected by marijuana use. This may raise new questions regarding the role of cannabinoids in the mononuclear phagocyte system.
**Fridman et al., 2023 (** 25)	All participants were SZ patients, divided into two groups of cannabis users and non-users	A CBC was obtained from first blood sample the morning after hospitalization. NLR was calculated.	NLR and MCV did not significantly differ between the two groups. Level of cannabis use did not significantly alter the results.	Inability to clinically assess the effects of cannabis use on symptom domains in SZ patients Inability to account for the wide range of antipsychotics given to the patients	No pathophysiologic substrates were discussed in this article.
**Alhassan et al., 2023 (** 26)	Participants were non-institutionalized US civilians divided into two groups current and never users of cannabis	Blood samples were obtained following a standardized protocol with a 12-hour fasting period before sampling	NLR was distributed similarly between the two groups, without a dose-response relationship with the frequency of cannabis use	Use of self-reported measures for marijuana and other substance use (risk of recall bias) Cross-sectional design Data gap in terms of dose and route of marijuana administration	No relevant pathophysiology discussed

### 2.5 Quality assessment

We utilized the Newcastle–Ottawa Quality Assessment Scale to assess bias risk in the cohort and cross-sectional studies included in our analysis. Case-control studies were categorized as having either a low (≥7 stars), moderate (5–6 stars), or high risk of bias (≤4 stars), with an overall quality score of 9 stars.

Two investigators, AN and MM, independently conducted the quality assessment, and any disparities were resolved through discussion and consensus. If necessary, a third investigator, RM, was involved in the resolution process.

### 2.6 Quantitative analysis

Weighted mean differences (WMDs) were employed to account for variations in NLR measurement methods across diverse studies. In our research, we utilized WMDs along with a 95% confidence interval (CI) to evaluate the disparities in NLR between cannabis users and control groups. The mean and standard deviation (SD) were computed based on the median, range, or interquartile range (IQR), following the methodology outlined by Wan et al. (27). The Cochrane Q-test and I2 index were utilized to assess between-study heterogeneity. It is important to note that, for the Cochrane's Q-test, a P value below 0.05 was considered statistically significant, and I2 values of 0.75, 0.50, and 0.25 indicated high, moderate, and low levels of heterogeneity, respectively. For certain secondary data, such as PLR, NLR, SII, BLR, and MLR, we used the original data for estimation. Furthermore, a random-effects model was employed for meta-analysis when dealing with heterogeneous results; otherwise, we utilized the random-effects model consistently. We conducted subgroup analysis based on the presence or absence of schizophrenia. Additionally, we evaluated publication bias by employing both the funnel plot and Egger's test, which measures the asymmetry in the funnel plot. Statistical analyses of the differences in NLR between cases and controls were carried out using STATA 17.0 (Stata Corporation, College Station, TX, USA). Unless stated otherwise, a P value less than 0.05 was considered statistically significant.

## 3 Results

### 3.1 Selection of studies

**Figure 1:**
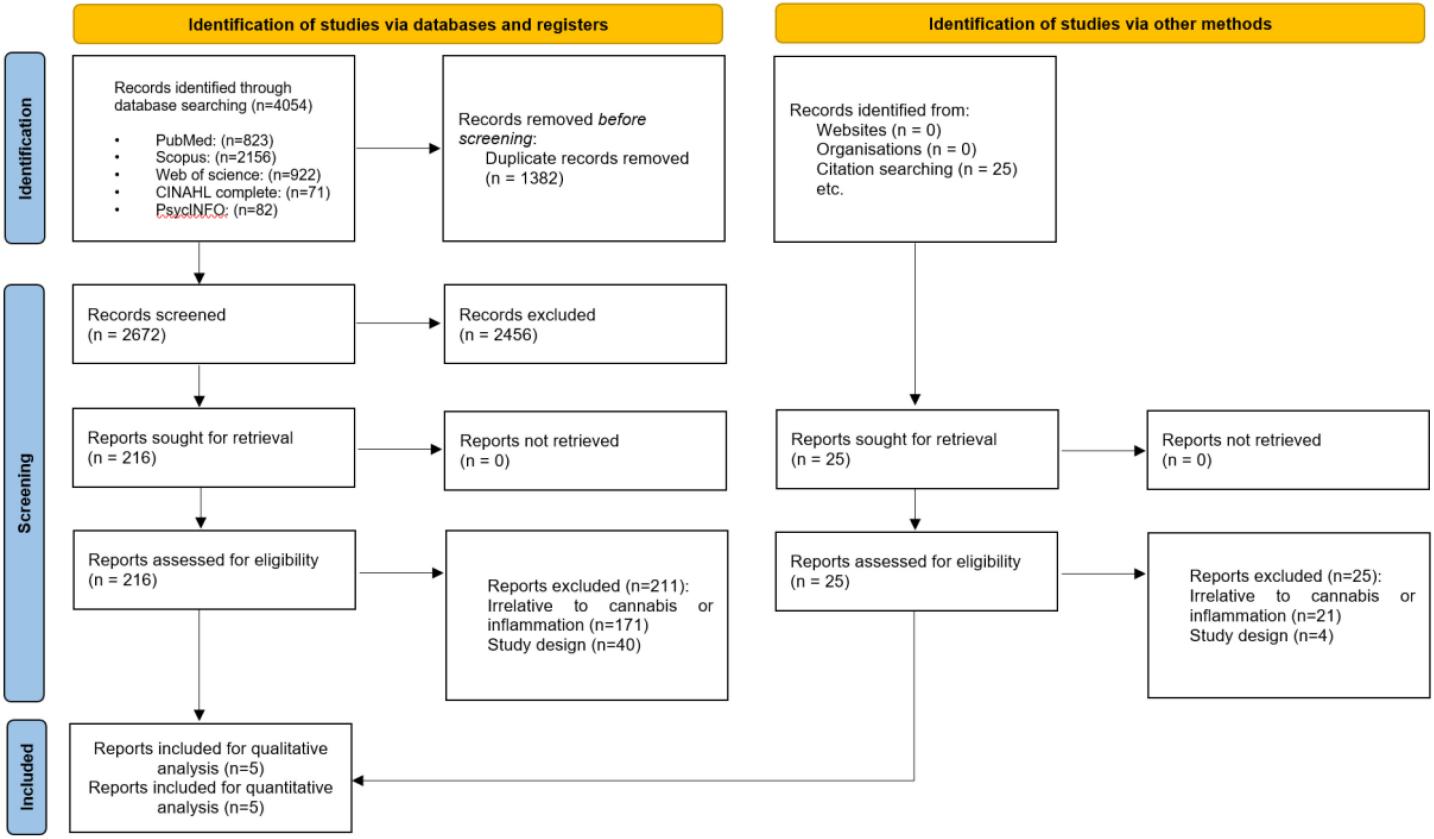
The Preferred Reporting Items for Systematic Reviews and Meta-Analysis (PRISMA) flow diagram of search results.

**Figure 1** illustrates the PRISMA flowchart. Initially, the search criteria generated 4054 articles. After eliminating 1382 duplicates using EndNote, we excluded 2672 articles following title and abstract screening. Subsequently, based on the eligibility criteria, we identified 216 articles as potentially relevant to our systematic review. Following a thorough evaluation of the full texts, 211 articles were excluded, resulting in five (22–26) articles remaining.

### 3.2 Study characteristics

A total of 5 studies (22–25, 28) were included in the study, 2 of which were conducted in Turkey (22, 24), one in Georgia (23), one in the US (26), and one in Israel (25). All studies had retrospective design and utilized electronic medical records to obtain the required data. In general, all studies delved into the chronic aspect of cannabis use and its effects on blood parameters. However, Alhassan et al. focused on patients with current cannabis use, defined as self-reported marijuana or Hashish use in the past 30 days (26).

Two studies (23, 25) investigated the effects of Cannabis use on blood parameters among patients diagnosed with schizophrenia. The former also utilized a within-subject design to further eliminate possible confounding factors that cannot be directly measured or conveniently estimated. Notably, NLR was not explicitly reported in the Goetz et al. study, indicating that the authors may not have primarily considered the immunomodulatory effects of cannabinoids as the major theme of their study. Alhassan et al. adjusted for psychiatric comorbidities(depression) and substance use (alcohol, tobacco, and other illegal substances) by means of inverse probability weighting (IPW), a previously well-described method for adjusting confounding baseline variables in observational studies. Both two other studies excluded subjects who were receiving treatment for a psychiatric condition or other inflammatory conditions that may alter the baseline for the outcomes (22, 24). In terms of demographics, only two of the studies (23, 26) have clearly mentioned the ethnic composition of the participants. Participants were exclusively male in one study (24). The others also had a high male-to-female ratio in both exposed and non-exposed groups (22, 23, 25).

Güzel et al. (22) found NLR to be significantly higher in cannabis users compared to nonusers (2.25 ± 0.99 vs 1.81 ± 0.61), while Orüm et al. (24) and Fridman et al. (25) found it to be insignificantly different. By contrast, in the Goetz et al. (22) study, NLR was significantly lower in cannabis users compared to nonusers (2.09 ± 3.6 vs 2.25 ± 2.71). Interestingly, Alhassan et al. found NLR means to be roughly the same in both current cannabis users and never users(2.1), with only a slightly different 95% CI (1.9-2.2 VS 2.0-2.1) (26).

All studies used a complete blood count (CBC) with differential to calculate NLR and other haematological markers of inflammation. Additionally, Guzel et al. (22) measured serum iron (SI), total iron binding capacity (TIBC), and unsaturated iron binding capacity (UIBC) and found the latter two to be significantly higher in chronic cannabis users compared to nonusers(345.70+-49.76 vs 284.52+-42.88 and 244.32+-57.36 vs 185.20+-59.93, respectively).

Most studies were constrained by limitations such as small sample size, lack of objective measures to confirm and quantify cannabis exposure, retrospective design, and other confounding variables that were not adjusted for (22–25). A discriminating feature of the Guzel et al. and Alhassan et al. studies is that smoking has been adjusted for as an important confounding variable (22, 26). Of note, Alhassan et al. studied a relatively large number of participants (3211 current users and 10213 never users of marijuana) sampled from the National Health and Nutritional Examination Survey (NHANES). They also adjusted for additional possible confounding variables such as lifestyle, health insurance status, and socioeconomic status (26).

None of the studies were primarily designed to reveal the underlying physiology of the immunomodulatory effects of cannabinoids. Nevertheless, Örüm et al. have discussed the possible role of cannabinoids in the mononuclear phagocyte system, considering that monocytes (a component of the mononuclear phagocyte system) were significantly lower in the cannabis user group in their study (24).

A summary of the general characteristics and main findings of the included studies is provided in **Tables 1**, **2**, respectively.

### 3.3 Risk of bias within studies

We evaluated the quality of all five included studies according to the Newcastle–Ottawa Quality Assessment Scale, and all five were of good quality. They had a low risk of bias (≥7 stars), as shown in **Table 1**.

### 3.4 Synthesis of results

#### 3.4.1 Overall results

**Figure 2:**
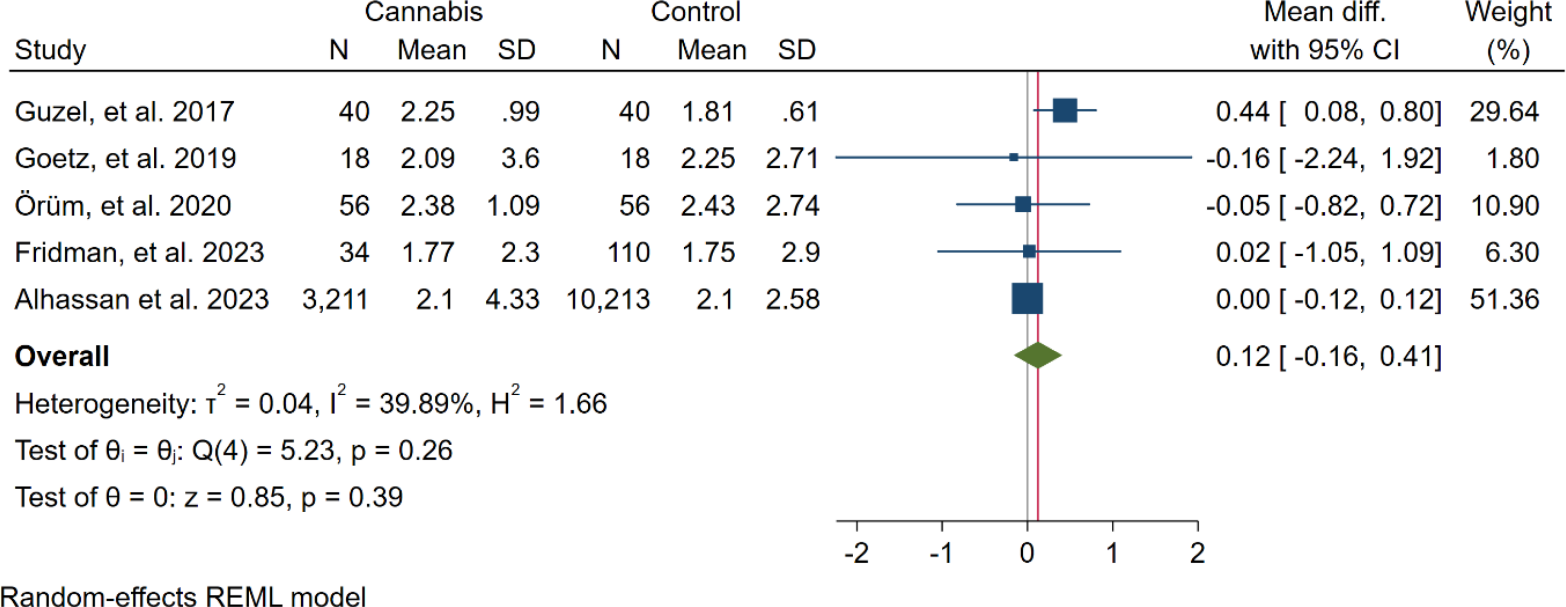
Forest plot of the overall result (NLR).

**Figure 3:**
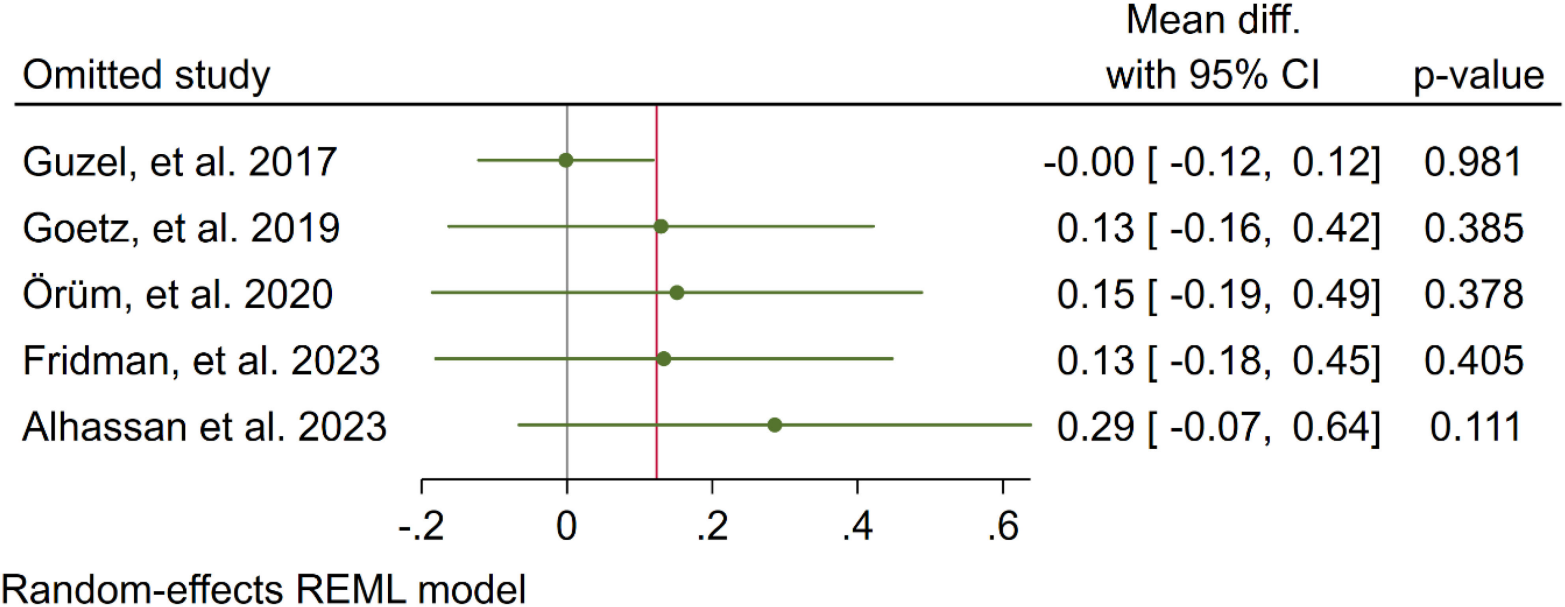
Sensitivity analysis of the overall result (NLR).

**Figure 4:**
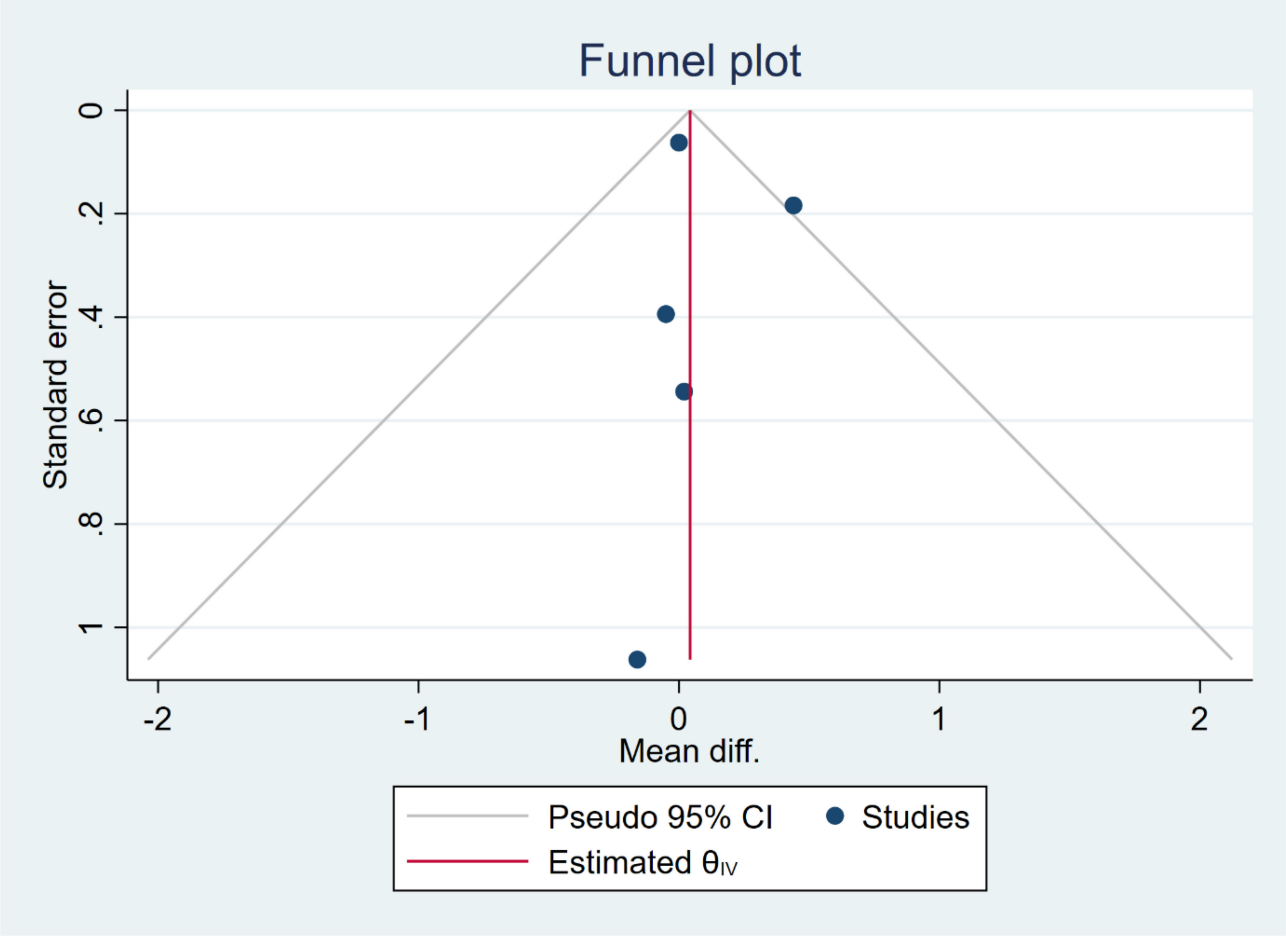
Funnel plot of the overall result.

In five studies, NLR levels were analysed in 3,359 participants who used cannabis and 10,437 participants who did not use cannabis. A non-significant difference in NLR was observed between the case and control groups (WMD: 0.12 [-0.16, 0.41], I^2^: 39.89%), as shown in **Figure 2**. To evaluate the individual impact of each study on the WMD, which is the primary outcome of our mathematical model, we conducted a sensitivity analysis by removing one study at a time. The results (**Figure 3**) showed that the omission of the Alcan et al. had a higher effect on pooled WMD but didn't change the direction (WMD: 0.29 [-0.07, 0.64]). We also assessed the presence of publication bias using Egger's test, Begg's test, and a funnel plot. The funnel plot exhibited a symmetric distribution of the data, indicating the absence of potential publication bias (**Figure 4**). Additionally, both Egger's and Begg's tests yielded low risk values (p = 0.84, p = 0.81, respectively) for publication bias.

**Figure 5:**
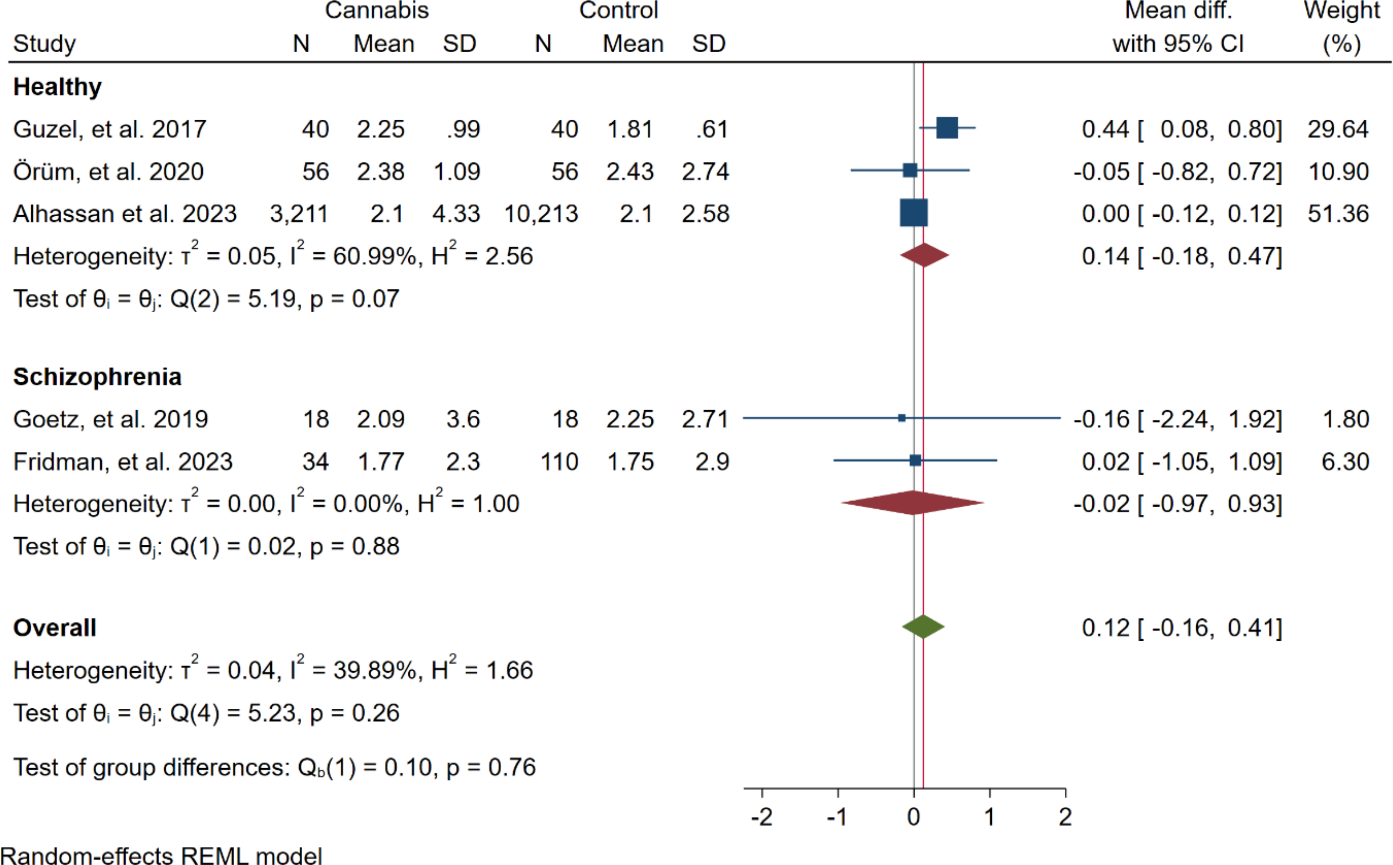
Subgroup meta-analysis of the overall results (NLR) based on the psychiatric condition of the participants.

Subgroup meta-analysis showed that there were no differences between NLR differences of case and control between studies performed on healthy (WMD: 0.14 [-0.18, 0.47]) and schizophrenia (-0.02 [-0.97, 0.93]) participants (p=0.76) (**Figure 5**).

#### 3.4.2 Secondary outcomes

In the secondary analysis, the differences in PLR, MPV, platelet count, neutrophil count, lymphocyte count, white blood cell count, monocyte count, basophil count, eosinophil count, SII, BLR, and MLR between case and control cohorts were assessed. Analysis showed that cannabis users had higher PLR (67.80 [44.54, 91.06]), neutrophil count (0.68 [0.25, 1.12]), white blood cell count (0.92 [0.43, 1.41]), monocyte count (0.11 [0.05, 0.16]), and SII (83.48 [5.92, 157.04]), compared to non-users. (**Table 3**; **Supplementary Material 2, Supplementary Figures 1-12**).

**Table 3:** Meta-analysis of secondary outcomes.

Variable	Number of studies	Number of participants		Weighted mean difference [95% CI]	P-value	I square (%)
		Case	Control			
**PLR**	3	130	206	67.80 [44.54, 91.06]	**<0.001**	0.00
**MPV**	3	130	206	-0.33 [-1.32, 0.67]	0.52	90.98
**Platelet count**	3	130	206	5.82 [-6.65, 18.28]	0.36	0.00
**Neutrophil count**	4	148	224	0.68 [0.25, 1.12]	**<0.001**	12.23
**Lymphocyte count**	4	148	224	0.05 [-0.10, 0.20]	0.52	0.00
**White blood cell count**	4	148	224	0.92 [0.43, 1.41]	**<0.001**	0.00
**Monocyte count**	4	148	224	0.11 [0.05, 0.16]	**<0.001**	3.60
**Basophil count**	3	114	114	0.00 [-0.01, 0.01]	0.75	0.09
**Eosinophil count**	2	96	96	0.00 [-0.09, 0.08]	0.96	52.91
**SII**	3	130	206	83.48 [5.92, 157.04]	**0.03**	78.59
**BLR**	3	114	114	0.00 [-0.02, 0.02]	0.91	0.01
**MLR**	4	148	224	0.04 [-0.04, 0.13]	0.32	0.00

CI, confidence interval; PLR, Platelet-to-lymphocyte ratio; MPV, Mean platelet volume; SII, Systemic immune-inflammation index; BLR, Basophil-to-lymphocyte ratio; MLR, Monocyte-to-lymphocyte ratio. Bold values indicate statistically significant differences.

## 4 Discussion

Our meta-analysis of five studies failed to find a significant difference in NLR between cannabis users and non-users (WMD: 0.12 [-0.16, 0.41], I^2^: 39.89%). However, additional analysis of secondary hematologic outcomes revealed that cannabis use was associated with a significantly higher PLR (67.80 [44.54, 91.06]), neutrophil count (0.68 [0.25, 1.12]), WBC count (0.92 [0.43, 1.41]), monocyte count (0.11 [0.05, 0.16]), and SII (83.48 [5.92, 157.04]) compared to non-use. No evidence of publication bias was detected. Taken together, these findings suggest that cannabis use may induce systemic inflammation, as evidenced by alterations in leukocyte profiles and SII.

Cannabis has been a subject of growing interest in medical research, and its impact on inflammation is a topic of significance. While some components of cannabis, particularly certain cannabinoids, have demonstrated anti-inflammatory properties in laboratory studies, the overall effect of cannabis on inflammation can be complex (29). Chronic cannabis use has been associated with potential pro-inflammatory effects. Regular and heavy cannabis consumption may lead to increased levels of pro-inflammatory cytokines (30).

NLR is a hematological parameter with significant implications in the field of medicine. It is a simple yet informative metric that reflects the balance between two vital immune cell types: neutrophils, which are the first responders to infection and inflammation, and lymphocytes, the key players in the adaptive immune response (16). NLR has gained recognition as a potential biomarker for assessing the state of inflammation and immune activation in various medical conditions. NLR has found applications in diverse fields, serving as a prognostic indicator in cardiovascular diseases, assessing cancer-related inflammation, and more (31–34). It can also be used as an affordable prognostic index for patients with traumatic brain injury (35).

Within the included studies, there was notable variability in the observed NLR among cannabis users compared to non-users. Specifically, Guzel et al. reported higher NLR values in cannabis users, suggesting a potential influence of cannabis on immune function (22). In contrast, Alhassan et al., Fridman et al., Goetz et al., and Orum et al. did not identify any significant difference in NLR between the two groups in their respective studies (23–26). This contrast in findings highlights the complexity of the relationship between cannabis use and NLR, emphasizing the need for further research to explain the underlying mechanisms and factors that may contribute to these variations.

In a noteworthy study by Inangil et al., the dynamic relationship between acute cannabis intoxication and NLR was investigated. Their research revealed a significant increase in NLR levels during episodes of acute cannabis intoxication. Interestingly, as part of their findings, they observed that NLR levels tended to decrease upon discharge from the acute intoxication state (36). This observation suggests that the impact of cannabis on NLR may be transient and fluctuate with changes in intoxication status, highlighting the need for further investigation into the dynamics of NLR alterations in response to cannabis use. Specifically, whether acute and chronic cannabis use tends to use different mechanisms of altering human immune response remains an open question.

In our investigation, we observed significant variations in hematologic parameters among cannabis users. Notably, there were increases in the neutrophil count, WBC, monocyte count, SII, and PLR. These findings align with the study by Alshaarawy et al., in which heavy cannabis users displayed significantly elevated WBC counts. This emphasizes the impact of heavy cannabis consumption on immune parameters, with crude and age-sex-adjusted mean WBC counts notably higher among heavy cannabis users compared to non-users. Modest differences were also evident in neutrophil counts, further underlining the influence of cannabis use on these hematologic factors. Although heavy users displayed higher monocyte counts, statistical significance was not achieved at the adjusted level. Moreover, Guzel et al. found higher levels of mean corpuscular hemoglobin (MCH), red cell distribution width (RDW), mean corpuscular volume (MCV), unsaturated iron binding capacity (UIBC), and total iron binding capacity (TIBC) among synthetic cannabinoid users compared to non-users (22). Additionally, in ROC analysis, Orum et al. found that monocyte count had fair diagnostic accuracy in distinguishing cannabis users from non-users (24). These findings collectively suggest a potential link between cannabis use and alterations in immune-related hematologic parameters, warranting further exploration into the underlying mechanisms and clinical implications (37).

Furthermore, there is a noteworthy link between schizophrenia and elevated pro-inflammatory factors, such as C-reactive protein (CRP) and NLR. Emerging evidence indicates that individuals with schizophrenia often exhibit elevated levels of these pro-inflammatory markers, which aligns with findings from a meta-analysis by Karageorgiou et al., demonstrating a significant increase in NLR in schizophrenia patients (20). Similarly, Miller et al.'s meta-analysis revealed a significant increase in CRP levels among patients with schizophrenia, further strengthening the evidence for increased inflammation within this population (38). Several mechanisms contributing to this phenomenon have been proposed, including chronic activation of immune cells such as macrophages, T lymphocytes, and microglia, leading to the secretion of inflammatory cytokines such as Interleukin-2 (IL-2), IL-6, IL-10, interferon-gamma (IFN-γ), and IL-4 (39). Additionally, research has explored the role of autoantibodies in schizophrenia, including anti-NMDA receptor autoantibodies, which have been associated with psychotic symptoms in encephalitis (40, 41).

The included studies suggest possible mechanisms linking cannabis use to alterations in hematological parameters. Orüm et al. highlighted the role of the mononuclear phagocyte system, as monocytes were lower in cannabis users. The psychoactive component THC may suppress immune cell functions, such as monocyte chemotaxis (24). Guzel et al. posit that chronic cannabinoid use leads to increased neutrophils and monocytes, reflecting immunologic reactivity. They suggest that the effects of synthetic cannabinoids on CB2 receptors and contaminants during production could underlie inflammation (22). Fridman et al. noted cannabidiol's immunosuppressive actions, although schizophrenia's proinflammatory state may counter these (25). Further research is needed to fully characterize the complex, dose-dependent immunomodulatory effects of individual cannabinoids and the shifts between anti- and pro-inflammatory states.

Although cannabinoids are known to exert anti-inflammatory actions, our findings unexpectedly revealed elevated levels of inflammatory markers in chronic cannabis users. A potential explanation is that the immunomodulatory effects of cannabinoids are complex and dose-dependent. Low doses of cannabinoids have been shown to have anti-inflammatory properties mediated through cannabinoid receptors, including inducing apoptosis in inflammatory cells and suppressing pro-inflammatory cytokines (42). However, higher or chronic doses may lead to cannabinoid receptor downregulation and overexpression of inflammatory mediators. Additionally, the specific cannabinoid composition may influence the anti-inflammatory effects, as cannabidiol (CBD), cannabigerol (CBG), and CBD + tetrahydrocannabinol (THC) combinations appear to have more potent anti-inflammatory actions compared to THC alone *in vivo* (11). This differential effect of individual cannabinoids and their interactions could also help explain the pro-inflammatory changes seen in our heterogeneous sample of cannabis users. Thus, the longstanding debate of the pro or anti-inflammatory role of cannabinoids must be addressed according to the context in which the debate runs. While the results of our meta-analysis indicate an overall pro-inflammatory effect in chronic cannabis users, others may find contradicting results depending on the chronicity, dose, duration, and route of cannabis administration.

Cannabis use can affect immune function through multiple mechanisms. Cannabinoids, particularly THC, interact with cannabinoid receptors CB1 and CB2, which are expressed on immune cells (43). This interaction can suppress T-cell responses, reduce inflammatory cytokine production, and induce apoptosis in immune cells (42). Additionally, cannabis use may alter the gut microbiome, indirectly affecting immune function through the gut-immune axis (44). Chronic cannabis use has been associated with decreased production of proinflammatory mediators and increased anti-inflammatory cytokines, potentially leading to an overall immunosuppressive effect (45). Furthermore, cannabis smoke contains similar harmful compounds to tobacco smoke, which may cause oxidative stress and DNA damage in immune cells (46). However, certain cannabinoids, such as CBD, have shown potential anti-inflammatory properties, suggesting a complex relationship between cannabis and immune function (47). The net effect of cannabis on immune function likely depends on factors such as dosage, frequency of use, and individual genetic variations in cannabinoid metabolism and receptor expression (43, 45).

Cannabis use has been shown to significantly affect sleep patterns, which in turn can influence inflammatory markers such as NLR. Studies indicate that while some users may experience improved sleep, others report disturbances, particularly with long-term use (48). Sleep disturbances are known to exacerbate inflammatory responses, suggesting that the sleep-modulating effects of cannabis could indirectly impact NLR levels (49). Daytime sleepiness and obstructive sleep apnea have been shown to have positive associations with NLR (50, 51).

Recent studies have highlighted the complex interplay between cannabinoids and epigenetic mechanisms in modulating inflammation. Cannabinoids have been found to cause epigenetic changes through processes like DNA methylation, histone modifications, and microRNA regulation (52). These changes can significantly impact immune cell differentiation and function. For instance, THC has been observed to downregulate pro-inflammatory miRNA clusters while upregulating anti-inflammatory miRNAs, leading to increased production of regulatory T-cells and suppression of inflammatory cytokines (53–55). Additionally, cannabinoids induce DNA methylation changes in inflammation-related genes and alter histone marks associated with T-cell differentiation (56). While genetic factors likely influence individual responses to cannabinoids, combining cannabinoid treatment with other immunomodulatory therapies may enhance anti-inflammatory effects (57). Further research is needed to identify the specific genetic factors involved and optimize combination treatment strategies for managing inflammation in cannabis users.

Despite the compelling findings in our systematic review and meta-analysis, several limitations must be acknowledged. First, the number of included studies was relatively small, comprising only five studies. This limited sample size may limit the generalizability of our results. Second, there was heterogeneity in the observed NLR values among cannabis users in the included studies. Although we conducted sensitivity analyses and assessed publication bias, the potential influence of unmeasured confounding variables cannot be entirely ruled out. Finally, the cross-sectional design of all five studies included in this meta-analysis makes it extremely difficult to draw a firm conclusion regarding a causal relationship between cannabis use and elevated markers of systemic inflammation. Further longitudinal studies with larger sample sizes and more statistically robust analyses are required to better understand the impact of cannabinoids on human immune status.

## 5 Conclusion

In conclusion, our systematic review and meta-analysis provide valuable insights into the diagnostic value of NLR in cannabis users. The findings suggest that cannabis use is associated with alterations in NLR and other hematologic parameters, indicating potential effects on immune function. However, the relationship between cannabis use and NLR is complex, with findings varying across studies. While these results show possible immune effects of cannabis use, it's important to note that their long-term clinical implications remain unclear. The efficacy and adverse effects of cannabis on the immune system likely depend on various factors, including frequency and duration of use, method of consumption, and individual health status.

Additionally, our study highlights the need for further research to clarify the mechanisms underlying these alterations. Longitudinal studies are necessary to fully explain the long-term immune consequences of cannabis use, which is essential for informing public health policies and clinical guidelines. Furthermore, we discussed the link between schizophrenia and elevated pro-inflammatory factors such as CRP and NLR, emphasizing the broader implications of our findings for understanding the relationship between cannabis use, immune function, and mental health. Overall, our study contributes to the growing body of literature on cannabis-related immunological changes and underscores the importance of continued investigation in this area.

## Data Availability

The original contributions presented in the study are included in the article/**Supplementary Material**, further inquiries can be directed to the corresponding authors.
