# Supplementary material for: The association between hematological markers of inflammation and chronic cannabis use: a systematic review and meta-analysis of observational studies
Source: Front Psychiatry. 2024 Oct 22;15:1438002. doi: 10.3389/fpsyt.2024.1438002 (PMC11534734; doi:10.3389/fpsyt.2024.1438002)
Supplement: Supplementary file 1 [file DataSheet1.pdf]

Databases:

1. PubMed: 823 results

#1: ((((((("Cannabis"[Mesh] OR "Marijuana Abuse"[Mesh] OR "Medical Marijuana"[Mesh]) OR (Cannabi*[Title/Abstract])) OR (Marihuana*[Title/Abstract])) OR (cannabis[Title/Abstract])) OR (Hashish[Title/Abstract])) OR (Bhang[Title/Abstract])) OR (Ganja[Title/Abstract])) OR (Hemp[Title/Abstract])

#2: (((((((Neutrophil*[Title/Abstract]) OR ("Neutrophils"[Mesh])) OR (lymphocyte*[Title/Abstract])) OR ("Lymphocytes"[Mesh])) OR (Platelet*[Title/Abstract])) OR ("Plateletpheresis"[Mesh] OR "Blood Platelets"[Mesh])) OR ("neutrophil to lymphocyte ratio"[Title/Abstract])) OR (NLR[Title/Abstract])

Final: #1 AND #2

2. Scopus: 2156 results

#1: TITLE-ABS-KEY(Cannabi*) OR TITLE-ABS-KEY(cannabis) OR TITLE-ABS-KEY(marijuana*) OR TITLE-ABS-KEY(Hashish) OR TITLE-ABS-KEY(Bhang) OR TITLE-ABS-KEY(Ganja) OR TITLE-ABS-KEY(Hemp)

#2: TITLE-ABS-KEY(Neutrophil*) OR TITLE-ABS-KEY(lymphocyte*) OR TITLE-ABS-KEY(Platelet*) OR TITLE-ABS-KEY(“neutrophil to lymphocyte ratio”) OR TITLE-ABS-KEY(NLR)

Final: #1 AND #2

3. Web of Science: 922 results

#1: TS=(Cannabi* OR cannabis OR marijuana* OR Hashish OR Bhang OR Ganja OR Hemp)

#2: TS=(Neutrophil* OR lymphocyte* OR Platelet* OR “neutrophil to lymphocyte ratio” OR NLR)

Final: #1 AND #2

4. PsycINFO: 71 results

TI Cannabi* OR TI cannabis OR AB marijuana* OR TI Hashish OR TI Bhang OR TI Ganja OR TI Hemp OR AB Cannabi* OR AB cannabis OR marijuana* OR AB Hashish OR AB Bhang OR AB Ganja OR AB Hemp

TI Neutrophil* OR TI lymphocyte* OR TI Platelet* OR TI “neutrophil to lymphocyte ratio” OR TI NLR OR AB Neutrophil* OR AB lymphocyte* OR AB Platelet* OR AB “neutrophil to lymphocyte ratio” OR AB NLR

Final: #1 AND #2

5. CINAHL complete: 82 results

TI Cannabi* OR TI cannabis OR AB marijuana* OR TI Hashish OR TI Bhang OR TI Ganja OR TI Hemp OR AB Cannabi* OR AB cannabis OR marijuana* OR AB Hashish OR AB Bhang OR AB Ganja OR AB Hemp

TI Neutrophil* OR TI lymphocyte* OR TI Platelet* OR TI “neutrophil to lymphocyte ratio” OR TI NLR OR AB Neutrophil* OR AB lymphocyte* OR AB Platelet* OR AB “neutrophil to lymphocyte ratio” OR AB NLR

Final: #1 AND #2
