# Supplementary material for: The association between hematological markers of inflammation and chronic cannabis use: a systematic review and meta-analysis of observational studies
Source: Front Psychiatry. 2024 Oct 22;15:1438002. doi: 10.3389/fpsyt.2024.1438002 (PMC11534734; doi:10.3389/fpsyt.2024.1438002)
Supplement: Supplementary file 2 [file DataSheet2.pdf]

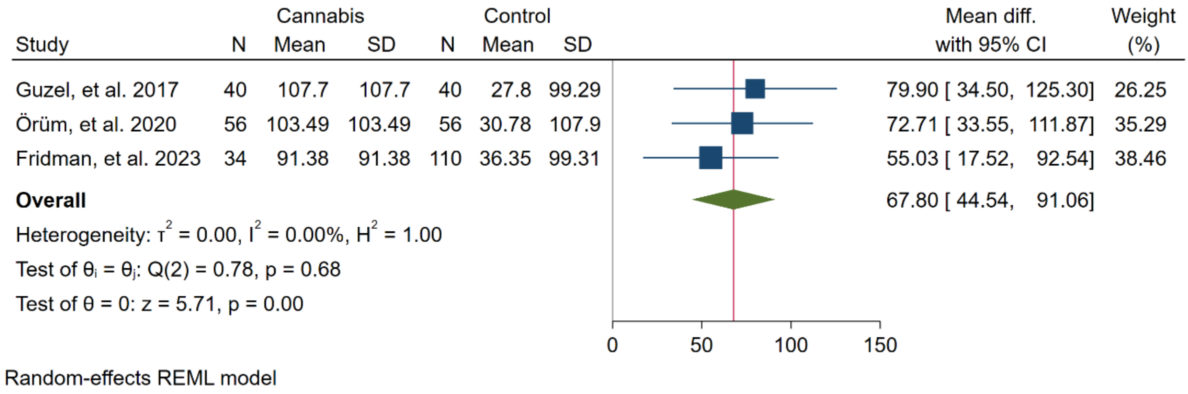

Figure S1. Forest plot of the overall result (PLR)

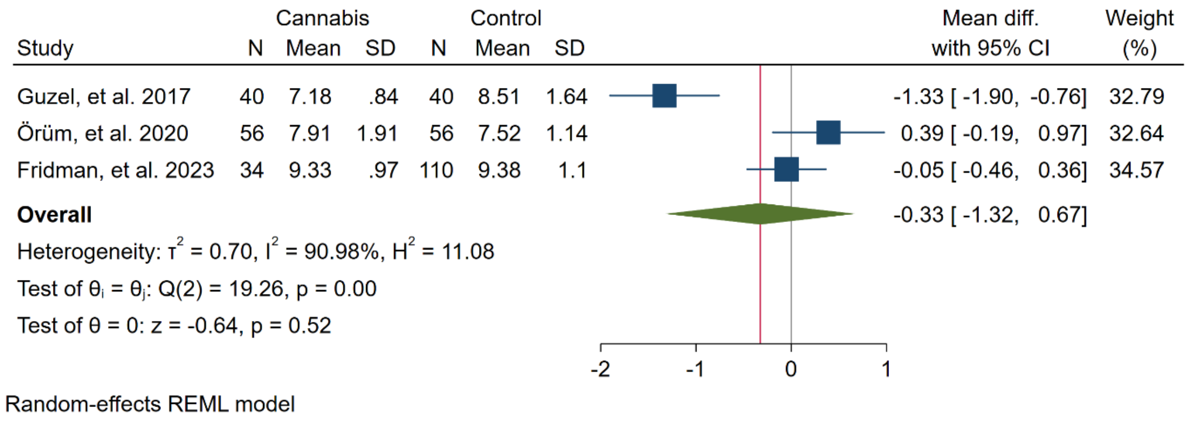

Figure S2. Forest plot of the overall result (MPV)

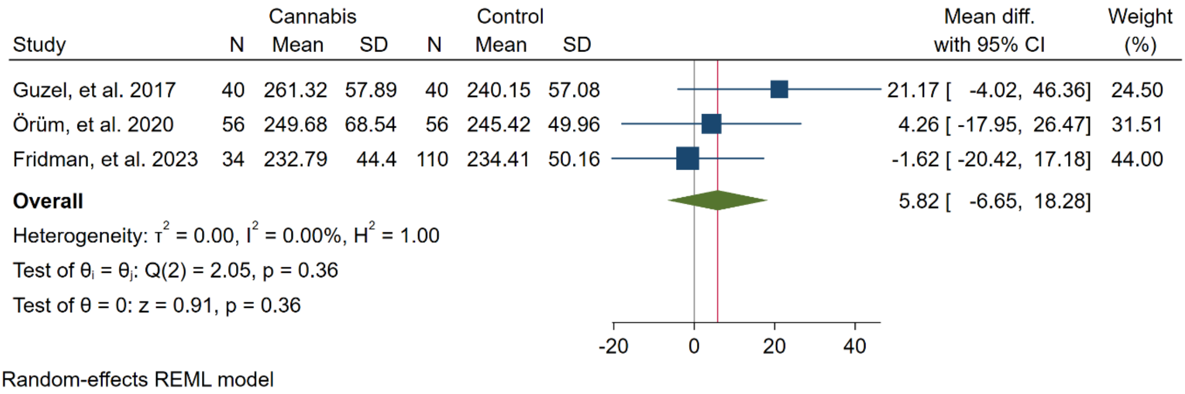

Figure S3. Forest plot of the overall result (Platelet count)

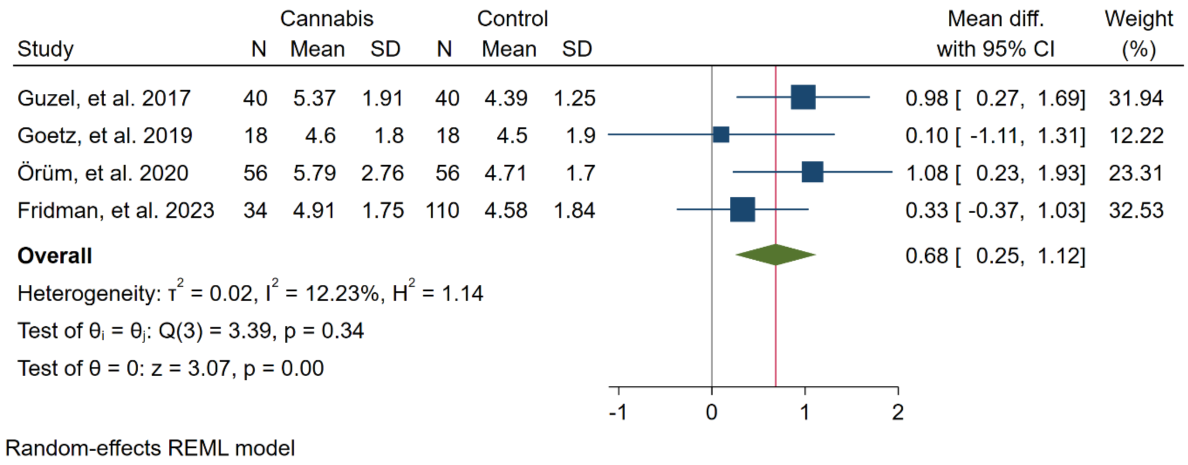

Figure S4. Forest plot of the overall result (Neutrophil count)

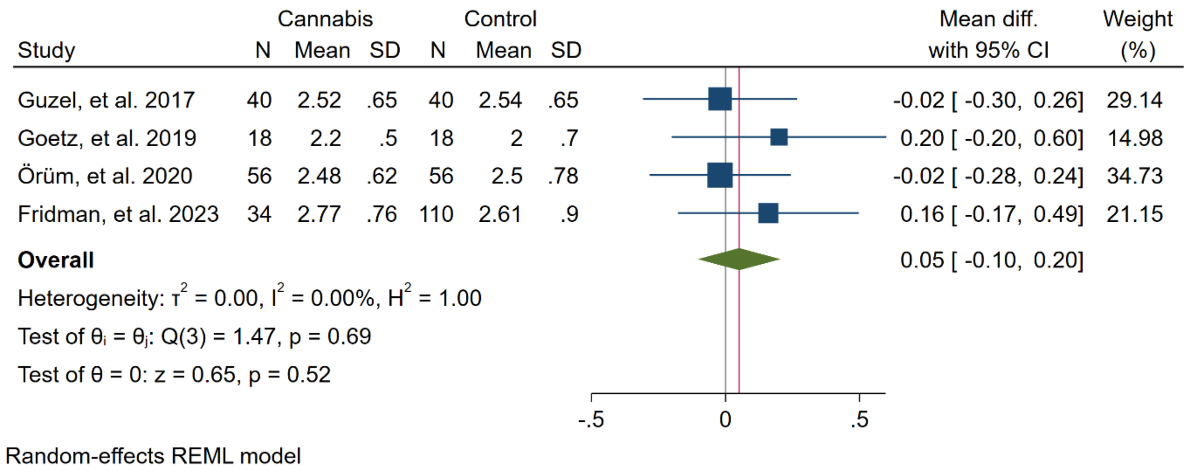

Figure S5. Forest plot of the overall result (Lymphocyte count)

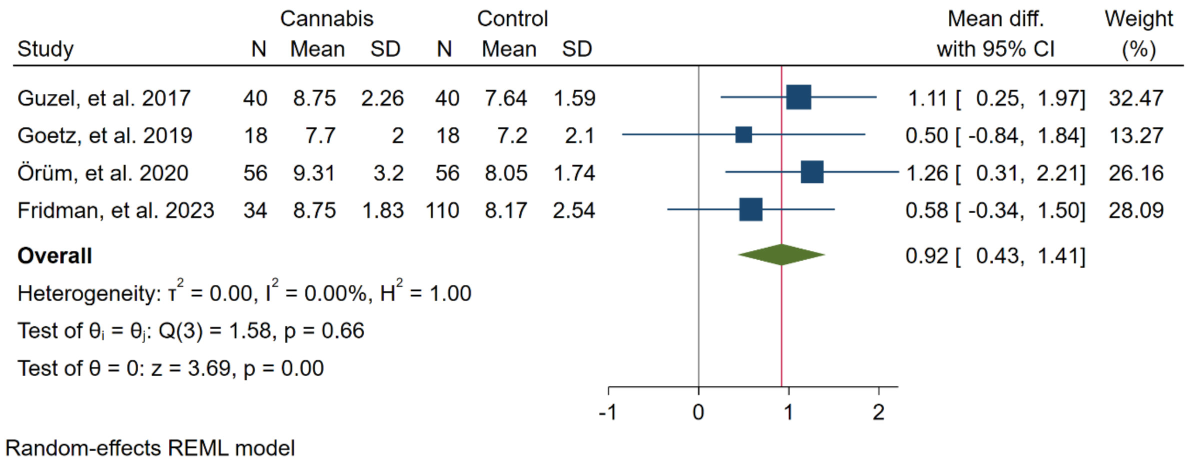

Figure S6. Forest plot of the overall result (White blood cell count)

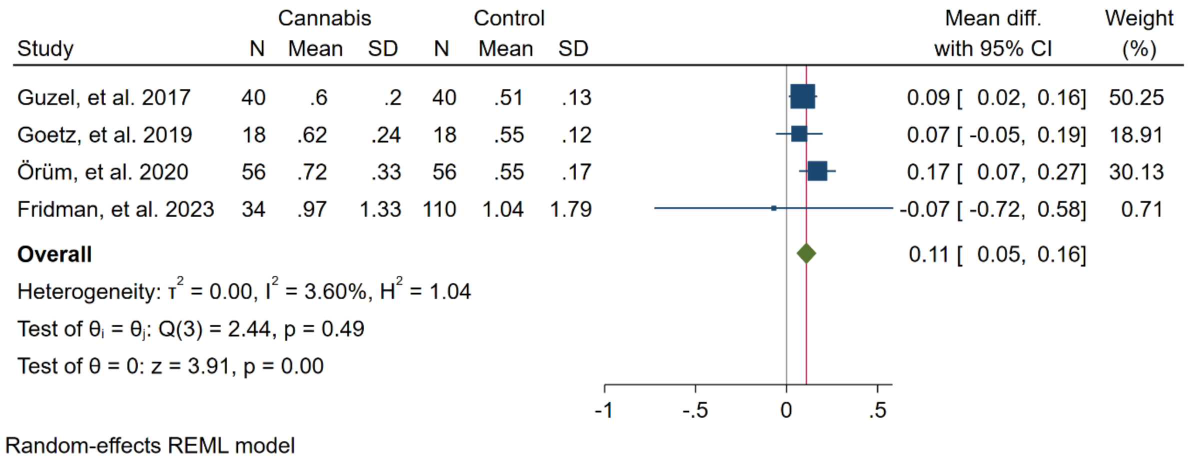

Figure S7. Forest plot of the overall result (Monocyte count)

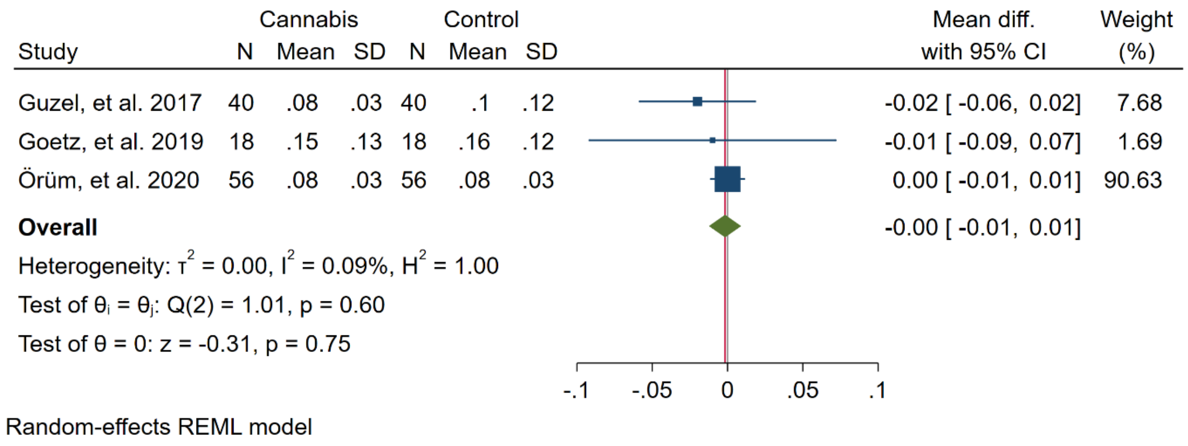

Figure S8. Forest plot of the overall result (Basophil count)

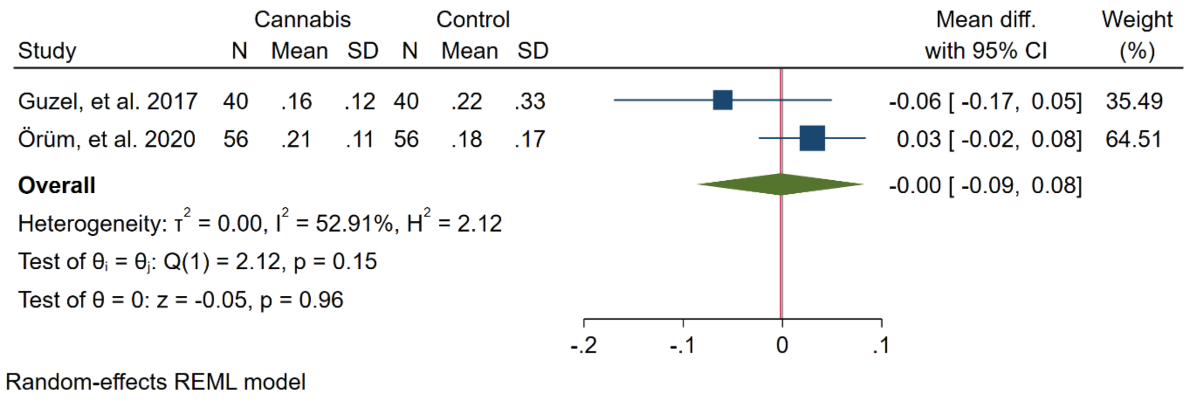

Figure S9. Forest plot of the overall result (Eosinophil count)

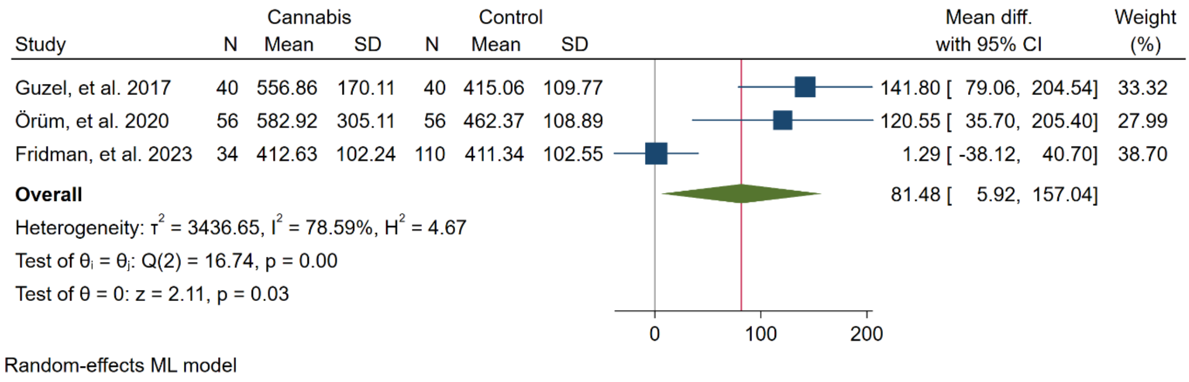

Figure S10. Forest plot of the overall result (Systemic immune-inflammation index (SII))

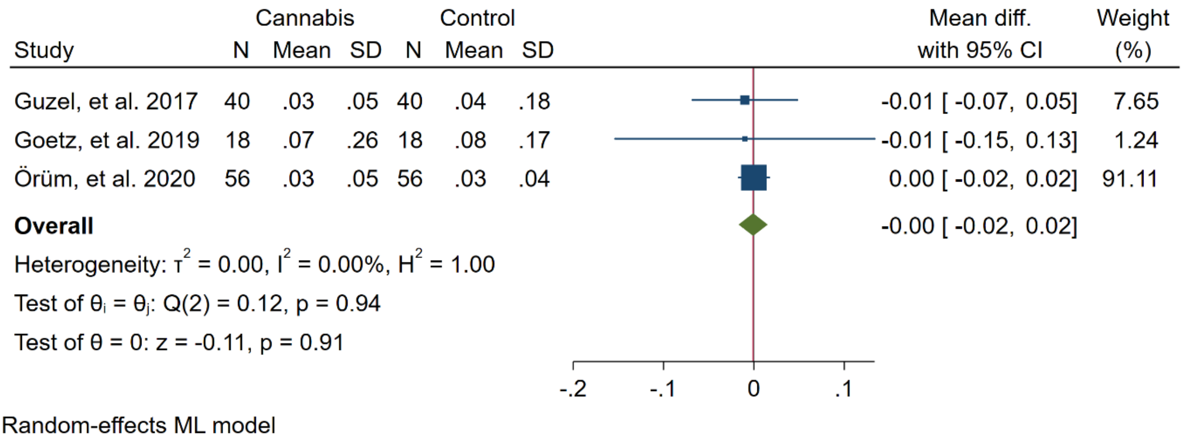

Figure S11. Forest plot of the overall result (Basophile to lymphocyte ratio (BLR))

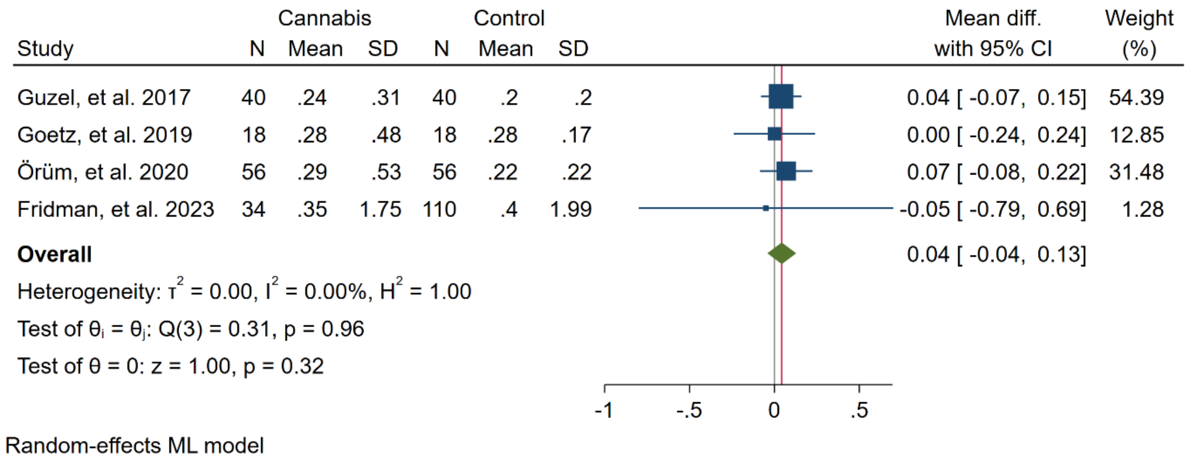

Figure S12. Forest plot of the overall result (Monocyte to lymphocyte ratio (MLR))
